# The frequency and nature of incidental findings in large-field cone beam computed tomography scans of an orthodontic sample

**DOI:** 10.1186/s40510-014-0037-x

**Published:** 2014-06-11

**Authors:** Ryan Edwards, Noura Alsufyani, Giseon Heo, Carlos Flores-Mir

**Affiliations:** Orthodontic Graduate Program, Faculty of Medicine and Dentistry, University of Alberta, Edmonton, T6G 2R3 Alberta Canada; Department of Dentistry, Faculty of Medicine and Dentistry, University of Alberta, Edmonton, T6G 2R3 Alberta Canada

**Keywords:** Cone beam computed tomography, Incidental findings, Maxillofacial region

## Abstract

**Background:**

The aim of this study is to evaluate the nature and frequency of incidental findings in large-field maxillofacial cone beam computed tomography (CBCT).

**Methods:**

A total of 427 consecutive CBCT radiologic reports obtained for orthodontic purposes were retrospectively reviewed. Findings were summarized and categorized into six anatomic categories.

**Results:**

A total of 842 incidental findings were reported in the 427 CBCT scans (1.97 findings/scan). The most prevalent findings were those located in the airway (42.3%), followed by the paranasal sinuses (30.9%), dentoalveolar (14.7%), surrounding hard/soft tissues (4.0%), temporomandibular joint (TMJ) (6.4%), and cervical vertebrae (1.3%) regions. Non-odontogenic findings, defined as those located outside the dentition and associated alveolus, represented 718 of the 842 (85.3%) findings.

**Conclusions:**

This study confirms the high occurrence of incidental findings in large-field maxillofacial CBCT scans in a sample of orthodontically referred cases. The majority are extragnathic findings, which can be normally considered outside the regions of interest of many dental clinicians. Specifically, incidental findings in the naso-oropharyngeal and paranasal air sinuses are the most frequent. This underscores the need for comprehensive review of the entire data volume and the requisite to properly document all findings, regardless of the region of interest.

**Electronic supplementary material:**

The online version of this article (doi:10.1186/s40510-014-0037-x) contains supplementary material, which is available to authorized users.

## Background

Cone beam computed tomography (CBCT) has been rapidly integrating into the field of dentistry to produce three-dimensional (3-D) imaging of the craniofacial complex. Current applications include, but are not limited to, specific orthodontic diagnosis, evaluation of the temporomandibular joint (TMJ), visualization of impacted teeth, evaluation of root resorption, preoperative implant planning, upper airway analysis, and presurgical treatment planning for both orthognathic surgery and craniofacial/cleft lip and palate cases [1–10].

When compared with conventional 2-D imaging, CBCT captures a much larger field of view. As such, there is an increased potential to identify incidental findings (IFs). IFs are defined as any and all discovered findings, detected by CT, MRI, CBCT, or any other imaging modalities that are unrelated to the clinical indication for the imaging being performed [11]. Arguably, as important as the detection is the action that each unexpected finding invokes, in terms of deciding the necessity for further evaluation and/or management [12]. As a large majority of IFs detected in CBCT imaging are extragnathic [13], the dental clinician may be unfamiliar with interpretation of anatomical structures outside the primary region of interest [14]. As such, the European Academy of Dento-MaxilloFacial Radiology (EADMFR) and the American Academy of Oral and Maxillofacial Radiology (AAOMR) outline that if the interpreting clinician is not highly experienced in CBCT interpretation, appropriate referral is required to an oral and maxillofacial radiologist (OMFR) for review and that the entire volume must be interpreted regardless of the region of interest [15, 16].

A number of studies in the literature have investigated the frequency of IFs in CBCT imaging in various patient samples [14],[17–23]. Of these, only two have investigated an orthodontic sample exclusively [17, 20]. Thus, additional studies are required to further define the nature of IFs in CBCT imaging in order to provide an accurate estimation of potential findings and pathologies, specifically in orthodontic patients. This descriptive study aims to assess the type, frequency, and location of incidental findings in large-field maxillofacial CBCT imaging, collected retrospectively via radiologic reports from an orthodontic sample.

## Methods

From a private diagnostic imaging center, 427 consecutive patients were retrospectively evaluated via chart review. No sample size calculations were performed. Instead, the chosen sample was deemed appropriate in size by comparison with similar studies in the literature. All patients received a single large field-of-view CBCT scan between the dates of 21 April 2011 and 21 May 2013, for the purpose of comprehensive diagnostic orthodontic records. All scans were acquired using an i-CAT Next Generation machine (Imaging Sciences International, Hatfield, PA, USA). Ethics approval for the retrospective chart review was obtained from the University of Alberta Health Research Ethics Board - Health Panel.

The kilovoltage (kV) and milliamperage (mA) were fixed (120 kV, 5 mA), but volume height, imaging time, and reconstruction voxel size varied slightly. All scans were acquired using a large field of view, which extended from the roof of the orbits inferiorly to at least the second cervical vertebrae. The voxel size ranged from 0.2 to 0.3 mm, with the vast majority (97.2% of scans) using a voxel size of 0.3 mm. The time of exposure for the scans was 4.8 s for 195 subjects, 8.9 s for 215 subjects, and 26.9 s for 17 subjects, after acquiring the scout image.

Following comprehensive interpretation of each scan by a single, board-certified oral maxillofacial radiologist, the same OMFR generated written radiologic reports for each image. All scans were reviewed by the OMFR using the imaging software InVivoDental 5.0 (Anatomage, San Jose, CA, USA). If the OMFR had any uncertainties or doubts regarding any of the findings, other OMFRs were contacted to seek a consensus-based opinion. The radiology reports followed a consistent format and contained a listing of all radiographic findings, which were used to tabulate the data in this study. If an additional reason for imaging was indicated (ie., investigation of a clinically detected impacted cuspid), the specific finding(s) was/were not considered as incidental. The subject's charts were not reviewed for any coincidence between systemic conditions and the findings. The radiologist was considered blinded to the objective of the present study, as at the time the radiologic reports were generated, it was not apparent that this data would be collected retrospectively for analysis.

A single researcher (RE), not associated with the imaging center, retrospectively reviewed the radiologic reports and tabulated all findings for descriptive analysis by entering data into formulated tables using Microsoft Excel. Decisions regarding the placement of the individual findings into the specific anatomic categories were performed via consensus of three researchers. If a subject had more than one finding for any given anatomic region, the total number of findings was recorded. For example, if a subject had both adenoid hyperplasia and concha bullosa, both were recorded as airway findings. The absence of third molars was not included as an incidental finding, as these teeth are commonly missing [24] or may have been previously extracted.

The complete data collection process was repeated by the single researcher, separated by a 60-day period. Intra-examiner agreement was assessed using the kappa statistic. Both age and sex of the patients were collected. Using a Bonferroni corrected *α* of 0.008 (0.05/6), a series of logistic regression analyses were performed to investigate if for any given age, the odds of identifying an incidental finding was different between sexes, for any of the six individual anatomic regions.

## Results

**Figure 1:**
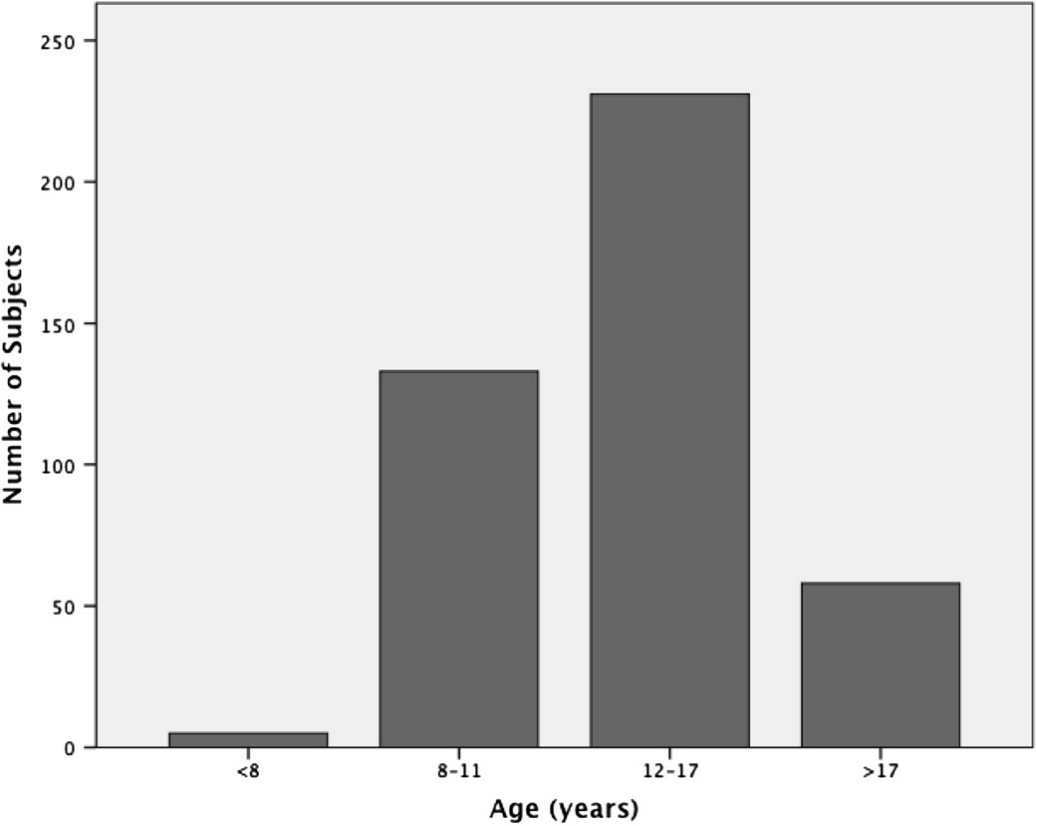
**Age distribution of orthodontic sample.**

Of the 427 subjects, 180 (42.2%) were males and 247 (57.8%) were females. The age of the patients who received scans ranged from 5 to 46 years; the mean age was 14.2 (±6.3) years and the median age was 12.0 years. The sample was divided into four age categories, aimed at representing subjects in the primary to early mixed dentition (<7 years), mid-mixed to early permanent dentition (8 to 11 years), adolescents in the permanent dentition (12 to 17 years), and adults (>17 years). The distribution of the total sample by age can be viewed in Figure 1.

**Figure 2:**
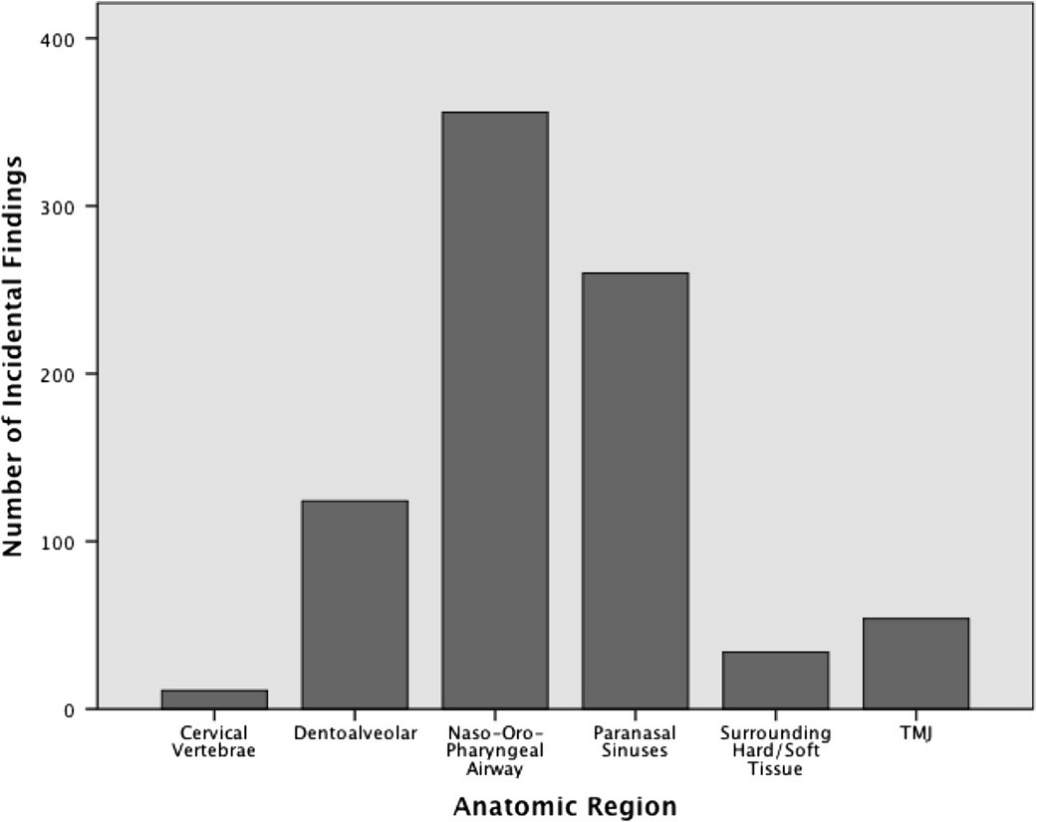
**Distribution of incidental findings among the six designated anatomic regions.**

All findings were categorized and placed into one of six common subgroups based on anatomic region. The groupings created for analysis were dentoalveolar, naso-oropharyngeal airway, paranasal sinuses, temporomandibular joint, cervical vertebrae, and surrounding hard/soft tissue. The groupings and frequency of individual findings can be viewed in Table 1 and Figure 2. With the exception of 23 patients in whom further investigation of suspected impacted canines was indicated in the clinical referral, no other additional clinical, radiographic, or histological information was used. For these 23 patients, the impacted canines were not included as incidental findings.

**Table 1:** **Frequency of incidental of findings among the 6 designated anatomic regions**

Incidental finding category	Frequency (***n***)	Percentage of IFs
**Cervical vertebrae**	11	1.3
Cervical vertebrae fusion	5	0.59
Cervical vertebral flattening	1	0.12
Cervical osteoarthritis	1	0.12
Mediolateral rotation (to L) of C2-C3 in relation to C1	1	0.12
Bony ossicle in C1-C2 region	1	0.12
Posterior ponticle of C1	2	0.24
**Dentoalveolar**	124	14.7
Supernumerary	6	0.71
Hypodontia (excluding third molars)	37	4.39
Microdontia	4	0.48
Impactions	28	3.33
Enamel pearl	1	0.12
Gemination	1	0.12
Retained primary tooth/fragment	6	0.71
Dilaceration	1	0.12
Severe root shortening (localized)	1	0.12
Ectopic position	2	0.24
Idiopathic osteosclerosis (DBI)	16	1.90
Odontogenic cyst	1	0.12
Simple bone cyst	2	0.24
Buccal bifurcation cyst	1	0.12
Torus mandibularis	1	0.12
Periapical cemento-ossesous dysplasia	2	0.24
External root resorption	5	0.59
Periapical rarefying osteitis	8	0.95
Periapical sclerosing osteitis	1	0.12
**Naso-oropharyngeal airway**	356	42.3
Choanal-retrochoanal polyp	1	0.12
Meatal obliteration	1	0.12
Adenoid hypertrophy	154	18.3
Lingual tonsil hypertrophy	55	6.53
Palatine tonsil hypertrophy	8	0.95
Concha bullosa	30	3.56
Nasal mucosal thickening; rhinitis	25	2.97
Nasal septal deviation	45	5.34
Nasal septal deviation (with bone spur)	22	2.61
Turbinate hypertrophy	5	0.59
Nasal polyps	1	0.12
Irregular soft tissue border of naso-oropharynx	2	0.24
Dystrophic calcification of tonsils	5	0.59
Concha enlargement	1	0.12
Opacification of the middle and superior nasal meatuses	1	0.12
**Paranasal sinuses**	260	30.9
Localized inflammatory conditions (mucositis-sinusitis)	152	18.1
Pansinusitis	11	1.31
Ostia blockage	11	1.31
Retention pseudocyst	58	6.89
Sinus hypoplasia	14	1.66
Sinus pnuematization	11	1.31
Sinus aplasia	1	0.12
Accessory ostia	1	0.12
Antrolith	1	0.12
**Surrounding soft/hard tissues**	34	4.0
Osteoma	1	0.12
Fibrous dysplasia	5	0.59
Jugular bulb pseudolesion	4	0.48
Pnuematization of mastoid air cells	12	1.43
Enlarged sella turcica	3	0.36
Soft tissue polyp	1	0.12
Ventriculoperitoneal shunt	1	0.12
Pineal gland calcification	1	0.12
Osteoma cutis	1	0.12
Calcified stylohyoid ligament	2	0.24
Dystrophic calcification of lymph node	1	0.12
Enlarged incisive (naso-palatine) canal	1	0.12
Depression/notch along the anterior surface of clivus	1	0.12
**Temporomandibular joint**	54	6.4
Condylar hypoplasia	16	1.90
Physiologic remodeling (flat margins, subchondral sclerosis)	19	2.26
Degenerative changes (osteophytes, erosions)	18	2.14
Bifid condyle	1	0.12

A total of 842 incidental findings were identified in 356 of the 427 scans (83.4%), representing an overall rate of 1.97 incidental findings per scan. The most common number of incidental findings per scan was 2, which occurred in 117 of 427 scans (Table 2). Non-odontogenic findings, defined as those located outside the dentition and associated alveolus, represented 718 of the 842 (85.3%) findings.

**Table 2:** **Incidental finding frequency categorized by number**

Number of incidental findings	Frequency (***n***)	Percentage of total
0	71	16.6
1	109	25.5
2	117	27.4
3	65	15.2
4	34	8.0
5	21	4.9
6	8	1.9
7	1	0.2
8	1	0.2

The most frequently identified incidental findings were those located in the naso-oropharyngeal airway, representing 42.3% of all findings. The second most common form of incidental findings was those identified in the paranasal air sinuses, representing 30.9% of all findings. Dentoalveolar findings represented 14.7%, while TMJ findings represented 6.4% of all incidental findings. Findings in the surrounding hard/soft tissues and cervical vertebrae represented 4.0% and 1.3%, respectively.

The kappa score measuring the level of inter-examiner agreement in the data collection was 1.0, indicating perfect agreement. The results of the logistic regression analysis suggest that when controlling for age, only one anatomic category demonstrated statistically significant differences between males and females (Table 3), where females were 2.55 times (*P* < 0.001, 95% CI [1.29,5.03]) more likely to have a TMJ finding than men.

**Table 3:** ***P*** **values obtained from a series of logistic regression analyses for each of the six anatomic regions**

Anatomic region	Sex (***P***value)
Dentoalveolar	0.447
Naso-oropharyngeal airway	0.556
Paranasal sinus	0.416
Temporomandibular joints	<0.001
Surrounding hard/soft tissues	0.144
Cervical vertebrae	0.808

*α = 0.05/6 = 0.008.

**Figure 3:**
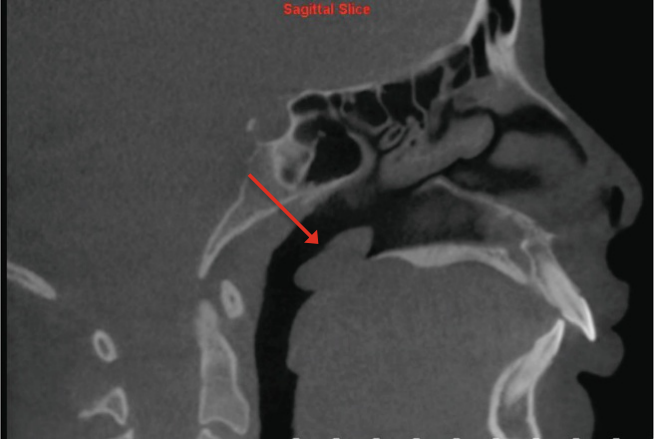
**Polypoidal soft tissue mass on superior surface of soft palate as viewed on sagittal slice.**

**Figure 4:**
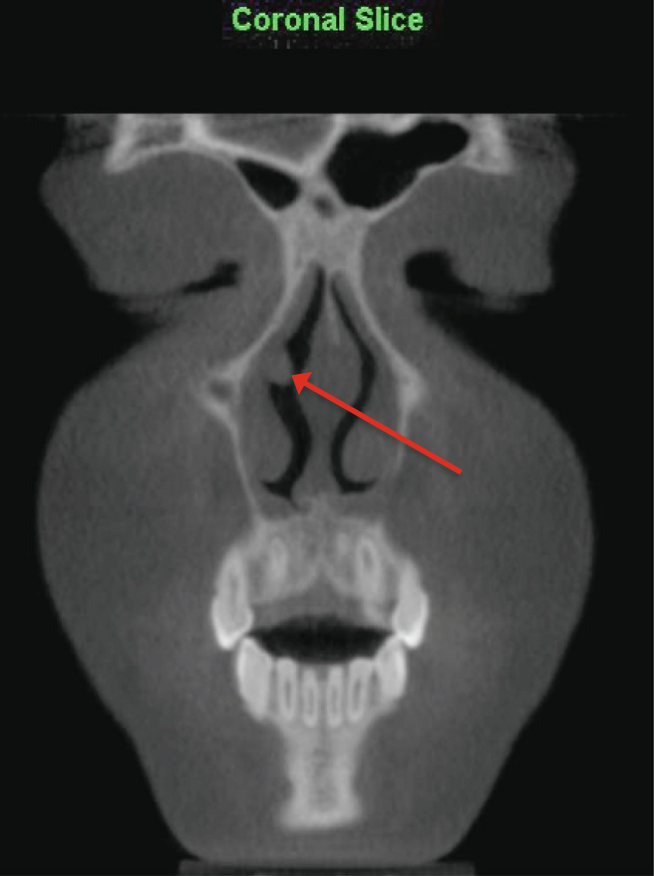
**An irregular thickening of the nasal cavity as viewed on coronal slice.**

**Figure 5:**
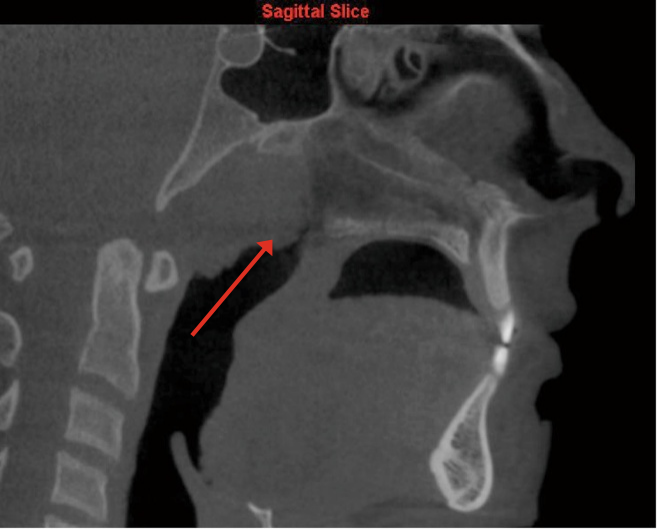
**Severe adenoid hypertrophy affecting patency of the nasopharyngeal airway as viewed on sagittal slice.**

**Figure 6:**
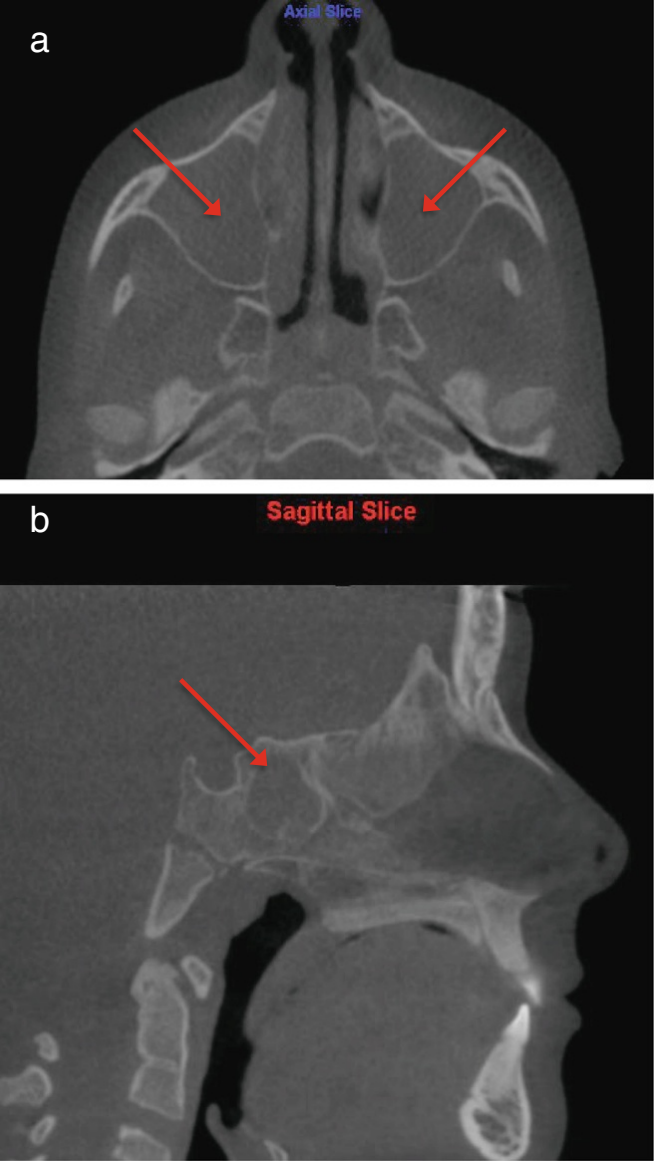
**Complete obliteration of maxillary, sphenoid, frontal, and ethmoid sinuses with soft tissue/mucosal-like density.** Viewed on **(a)** axial and **(b)** sagittal slices.

**Figure 7:**
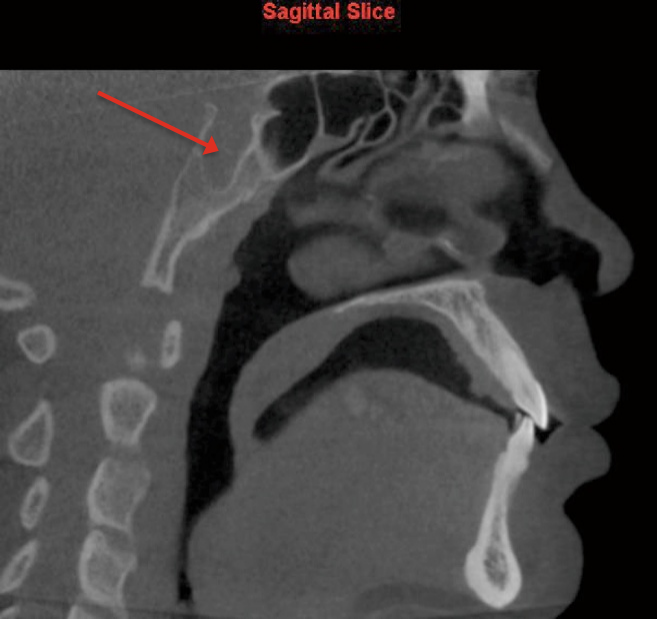
**Enlarged sella turcica as viewed on sagittal slice.**

**Figure 8:**
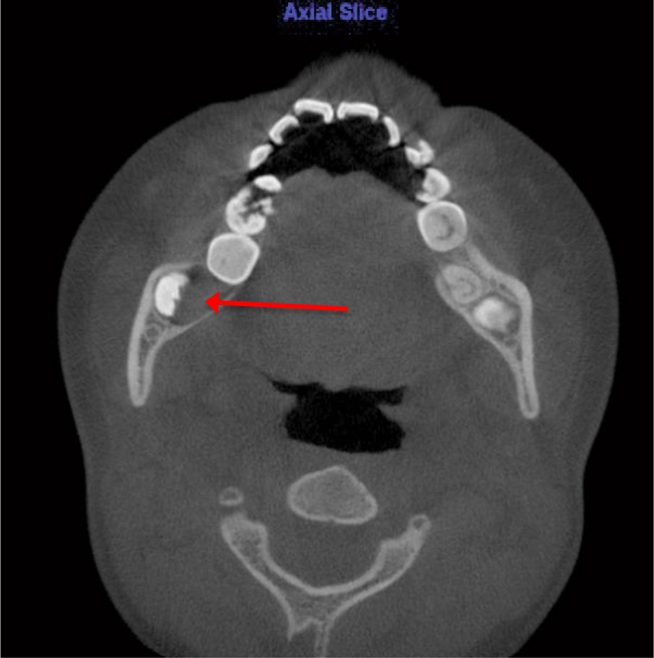
**Odontogenic cyst pericoronal to tooth 48 as viewed on axial slice.**

**Figure 9:**
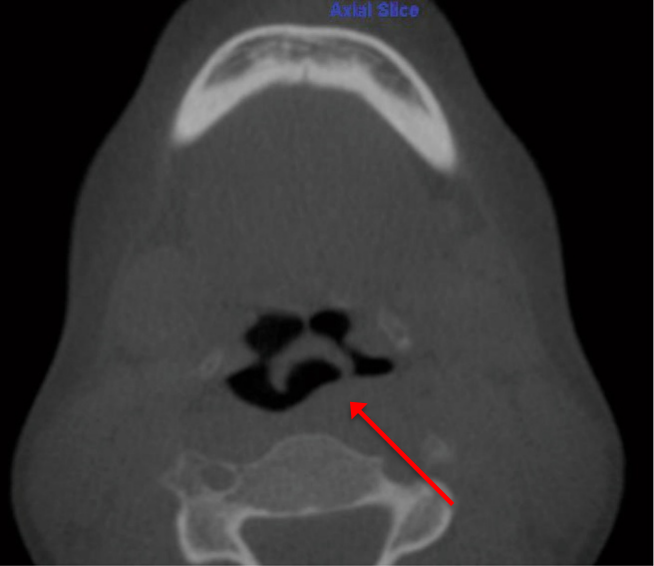
**Soft tissue asymmetry with thickening and enlargement of left-side pharynx and larynx as viewed on axial slice.**

Further follow-up was specifically suggested by the interpreting OMFR for the following seven findings:

1. Polypoidal soft tissue mass on the superior surface of soft palate (Figure 3)

2. An irregular thickening of the nasal cavity; nasal polyps cannot be ruled out (Figure 4)

3. Severe adenoid hypertrophy affecting patency of nasopharyngeal airway (Figure 5)

4. Complete obliteration of maxillary, sphenoid, frontal, and ethmoid sinuses with soft tissue/mucosal-like density (Figure 6)

5. Enlarged sella turcica (Figure 7)

6. Odontogenic cyst pericoronal to tooth 48 (Figure 8)

7. Soft tissue asymmetry with enlargement of the left-side pharynx and larynx (Figure 9)

## Discussion

CBCT imaging is increasingly being utilized in diagnosis and treatment planning in orthodontics. In this study, 427 consecutive CBCT radiologic reports of orthodontic patients were retrospectively reviewed from a private diagnostic imaging center. Reported findings include developmental findings, normal anatomic variants, age-related findings, and pathological findings. As mentioned in a previous systematic review [13], at least two methods for reporting the incidence of incidental findings are described in the literature: (i) by describing the absolute number of IFs detected or (ii) describing the number of CBCT scans that contain IFs. The former method, using the absolute number of IFs, is favored because it is highly likely for multiple IFs to be detected in a single CBCT scan; our results confirm this.

From our sample of orthodontic patients, a total of 842 IFs were identified in the 427 scans, representing an overall rate of 1.97 IFs per scan. It is known that the frequency of IFs in CBCT imaging varies among studies in the literature, ranging from 1.1 to 2.9 IFs per CBCT scan [14],[17–21]. The IF rate reported from our sample is thus similar to that of other studies. At least one IF was identified in 356 of 427 scans (83.4%), which is also similar to that of other studies in the literature, which report the number of CBCT scans containing at least one IF to be between 90.7% and 94.3% [14, 18, 21, 23]. However, in studies by Rheem et al. [22], Pliska et al. [17], Rheem et al. [22], and Cha et al. [19], it was respectively reported that IFs were identified in only 66%, 65.5%, 40.1%, and 24.5% of CBCT scans, which is significantly less than in our sample. These observed variations in the literature can likely be attributed to differences in the samples, such as age groups; in radiologist's reporting styles; and in the definition of the term incidental finding.

### Naso-oropharyngeal airway

The most common location for identified IFs was in the nasal-oropharyngeal airway, representing 42.3% of all findings, with adenoid hyperplasia (18.3%), nasal septal deviations (8.0%), and lingual tonsil hyperplasia (6.5%) identified most frequently. This high rate of airway findings is consistent with the literature, as various other CBCT studies have demonstrated that airway findings represent 8.4% to 35.0% of total CBCT findings [14],[17–19, 21, 23].

Septal deviations represented 8.0% of findings in our sample, which is less than the 19.4% reported by Smith et al. [25]. Concha bullosa, a common anatomical variation of the sino-nasal anatomy characterized by pneumatization of the nasal turbinates, represented 3.6% of findings. This is much less than other reports in the literature, in which the prevalence of concha bullosa varied from 35% to 68% [25–28]. The joint incidence of septal deviation and concha bullosa has been previously reported to be high (19.5% to 44.6%) [25, 29]. In our sample, of the 30 findings of concha bullosa, septal deviations were also identified in nine of these subjects (30%).

The majority (25.8%) of upper airway findings in our sample were due to varying forms of adeno-tonsillar hypertrophy, specifically adenoid/pharyngeal (18.3%), lingual (6.5%), and palatal tonsil (0.1%) hypertrophy. Upper airway obstruction has been described as a possible environmental cause of malocclusion and disharmonious dento-facial development observed in growing subjects [30–32]. Various studies have discussed the contributing role of not only adeno-tonsillar hypertrophy [33–35], but also nasal septum deviation [35, 36], allergic rhinitis [37], and inferior turbinate hypertrophy [38, 39] in partial upper airway obstruction.

The high frequency of airway findings in our sample demonstrates that CBCT can be an important tool in screening for airway abnormalities. However, the current reference standard for assessing the nasal cavity and nasopharynx remains nasoendoscopy (NE) [40]. An important distinction must be made between identifying potential upper airway constriction in CBCT imaging and relating it to the actual presence and/or severity of clinical obstruction. Specifically regarding adenoid size, it has been demonstrated in a recent study that CBCT imaging demonstrated excellent sensitivity (88%) and specificity (93%) when compared with NE [41]. In addition, the assessment of adenoid size using CBCT had strong accuracy (intra-class coefficient (ICC) = 0.80, 95% CI ±0.15) and very good inter-rater (ICC = 0.85, 95% CI ±0.08) and intra-rater reliability (ICC = 0.84, 95% CI ±0.08) among subjects. This suggests that CBCT can be a reliable and accurate tool for identifying adenoid enlargement. Similar studies should be conducted to investigate the sensitivity and specificity of CBCT compared to NE in regard to other airway findings, such as septal deviation and turbinate hypertrophy.

Despite the validation of CBCT for adenoid assessment, management decisions should be made on the basis of clinical history and NE, rather than entirely on radiologic findings [42]. Furthermore, CBCT imaging should never be considered a replacement to NE. However, when available because it was indicated for other reasons, this imaging technology does provide orthodontic clinicians with an accurate and reliable tool for the assessment of adenoid size, facilitating screening for and early detection of adenoid enlargement and other potential airway problems [41].

### Paranasal air sinus region

Paranasal sinus changes represented 30.9% of all findings in our sample, which is similar to other CBCT studies, in which sinus changes have been commonly demonstrated ranging from 23.9% to 62.6% of findings [14, 18, 20, 21, 43]. Many studies using MRI and medical CT imaging also confirm a high prevalence of incidental sinus findings. Havas et al. [44], using CT, reported changes in one or more paranasal sinuses in up to 42.5% of asymptomatic patients. Diament et al. [45] identified maxillary and ethmoid sinus opacifications in 50% of a pediatric sample referred for cranial CT. Lim et al. [46] and Gordts et al. [47] respectively reported that 32.3% and 45% of pediatric subjects have sinus abnormalities in non-ENT MRI imaging.

Localized inflammatory conditions consisting of mucositis-sinusitis (18.1%) and retention psuedocysts (6.89%) were the most frequently identified sinus findings. Concerning sinus mucosal inflammation in CBCT imaging, it is known from the literature that it is a common finding identified in 15.0% to 55.1% of patients [14, 18],[20–23, 48]. For the purposes of this study, sinusitis was defined as the radiographically detectable thickening of the sinus mucosa. Findings were based entirely on radiographic appearance, as no clinical information was assessed. Pansinusitis, an inflammation of all the paranasal sinuses, was present in 11 subjects, representing 1.31% of findings. In 10 of 11 pansinusitis patients, other concomitant airway findings were also reported, including adenoid hypertrophy (six subjects), blocked ostia (two subjects), and maxillary sinus hypoplasia (two subjects). Maxillary mucous retention pseudocysts are identified as incidental findings in 2.9% to 16.4% of CBCT scans [14, 17, 20, 23]. They usually spontaneously regress or show no significant change in size over the long term and rarely lead to symptoms [49]. It is suggested that in the absence of associated complications, conservative monitoring is the appropriate management strategy.

In evaluating maxillary sinus abnormalities using 2-D panoramic imaging, Vallo et al. [50] identified mucosal thickening in 12% and mucous retention cysts in 7% of radiographs. Bondemark et al. [51] identified sinus mucosal thickening in 26.8% of panoramic radiographs. Thus, panoramic radiography does allow for the identification of sinus abnormalities. However, it may not be as reliable a method as CBCT based on the limitations of 2-D imaging: magnification, distortion, and superimposition [52]. It must be mentioned that the frequency of sinus mucosal thickening and retention cysts can vary due to odontogenic factors, age, gender, season, and presence of allergies [53, 54]. As with airway findings, the importance of careful clinical correlation must be stressed when interpreting 3-D images of the paranasal sinuses, since minor opacification is a common finding, even in asymptomatic subjects [55, 56]. 3-D imaging may provide information regarding the extent of the mucosal disease, but findings correlate poorly with clinical signs and symptoms [55–57]. Therefore, 3-D imaging may help to support a clinical diagnosis, but it should not be interpreted out of context.

### Dentoalveolar region

There were 118 incidental findings (14.7%) located in the dentoalveolar region, most commonly hypodontia (4.4%). As mentioned, missing or non-developing third molars were not included as hypodontia in this study because it would inflate the number of findings, as it has been shown that the third molars are the most common congenitally missing tooth, with one or more missing in 9% to 20% of individuals [24]. In addition, due to the large range in age of our sample, in some subjects the third molar tooth germs would not yet be visible, while in others they may have been previously extracted. In the literature, opinions vary on the second most commonly missing tooth [58]. Some investigators [59–62] believe that it is the maxillary lateral incisor, whereas others [63, 64] believe that mandibular second premolar agenesis has a higher incidence. In our sample, of the total 37 congenitally missing teeth, 24 were second premolars (4.39%), 11 were maxillary laterals (1.31%), and 2 were mandibular central incisors (0.24%). These rates are comparable but slightly less than rates described in the literature [65]. Other common dentoalveolar findings were dental impactions (3.33%), idiopathic osteosclerosis (1.90%), and supernumerary teeth (0.71%). All of these findings can be readily identified in traditional 2-D imaging [66]. However, CBCT offers the advantage of more accurate localization [67] and assessment of adjacent structures [68], both of which have the potential to impact management decisions [69].

### Temporomandibular joint region

There were 54 findings in the temporomandibular joint (TMJ) region, representing 6.4% of all findings. The main findings were physiologic remodeling (2.3%), degenerative changes (2.1%), and condylar hypoplasia (1.9%). According to logistic regression analysis, when controlling for age, females were 2.55 times more likely to exhibit TMJ IFs than men (*P* < 0.001, 95% CI [1.29,5.03]). This finding is commonly supported in the literature [14, 22, 70].

The decision was made to place condylar changes into two main categories, either physiologic remodeling or actual degenerative changes, even though the radiographic signs of mild degenerative joint disease (DJD) can be similar to those associated with joint remodeling. Isolated TMJ flattening and/or subcondral sclerosis was interpreted as physiologic remodeling, while condylar erosions and/or osteophyte formation was interpreted as active condylar degeneration (DJD). Pette et al. [14] and Allareddy et al. [21] reported higher rates of degenerative TMJ changes in patients receiving CBCT imaging primarily for dental implant assessment, identifying degenerative changes in 39.0% and 6.2% of patients, respectively, while other studies investigating orthodontic populations report degenerative TMJ changes in only 0.5% to 3.6% of subjects [19, 20]. It has been demonstrated that the progression and severity of TMJ osseous changes are increased with advancing age [70, 71]. This lower incidence of degenerative changes in our sample, and other orthodontic cohorts in the literature, is likely due to the nature of an orthodontic population, i.e., consisting primarily of adolescents.

### Cervical vertebrae region

Cervical vertebral findings represented only 1.3% of all CBCT findings, a similar prevalence to other CBCT studies examining orthodontic populations [17, 20]. This low rate of vertebral findings may be expected, given the low mean age of our orthodontic sample and variation in the number of vertebrae included in each of the scans. This is in contrast to CBCT studies by Pette et al. [14] and Allareddy et al. [21] which examined samples with much higher mean ages. In these studies, cervical vertebral findings were respectively identified in 47.8% and 9.7% of subjects, with the degenerative changes representing the main finding. Vertebral fusion was the most predominant finding in this region in our sample (0.6%). The prevalence as demonstrated in other studies is 0.4% to 0.7% with no sex predilection, with C2-C3 being the most common location [72]. Generally, patients are asymptomatic, but increasing age or injury may precipitate symptoms as discal tear, rupture of the transverse ligament, and odontoid process fracture are common consequences. In addition to vertebral fusion, other findings have been identified in CBCT studies, including osteoarthritis, clefts, subchondral cysts, and osteophyte formation [14, 20, 21]. Many abnormalities of the cervical spine do not manifest themselves symptomatically until young adulthood, and if progressive degenerative defects are identified early, this may aid in the mitigation of the severity of their consequences [73]. With CBCT, the orthodontist and/or OMFR may be the first person to detect them and thus serve to screen and to refer for further assessment.

In our orthodontic sample, of the 842 reported findings, 718 (85.3%) were located in extragnathic locations (i.e., outside the dentition and alveolus). This is comparable to similar CBCT studies in the literature. Price et al. [18], in a sample of 300 consecutive patients, reported a total of 881 incidental findings, with 775 (88.0%) of these being extragnathic. In a sample of 318 dental implant patients, Pette et al. [14] reported that 93.7% of subjects had incidental extragnathic findings. They also identified both vascular and intracranial findings that were not reported in our sample. Internal carotid artery (ICA) calcifications were reported in 23.6% of their subjects and pineal gland calcification in 19.2% [14]. ICA calcifications were also identified in 5.7% of CBCT subjects by Allareddy et al. [21]. Similarly, ICA calcifications in CBCT were identified in 4.8% of subjects by Price et al. [18]. These findings were likely not identified in our sample due to major differences in mean age, as advanced age has been demonstrated to be a major risk factor for ICA calcification [74]. In panoramic imaging of large samples, Bayram et al. [75] and Kumagai et al. [76] respectively reported that ICA calcifications were identified in 2.1% and 4.0% of subjects. However, the presence of ICA calcifications does not always imply stenosis. The gold standard for the diagnosis of carotid artery stenosis (CAS) is duplex ultrasound and is utilized in cases of suspected CAS [77]. A number of studies have compared the incidence of ICA calcifications identified on panoramic radiography to CAS [77–80]. These studies, investigating populations over the age of 55, have observed positive ICA calcification in 2% to 5% of images [78–81]. Therefore, panoramic radiographs appear to be a valuable screening tool for CAS. However, due to the advantages of CBCT imaging (i.e., lack of overlapping structures, submillimeter voxel resolution, etc.), it may result in superior and more accurate screening for CAS. The relationship between ICA calcifications identified in CBCT imaging and CSA identified in duplex ultrasound must be further evaluated.

The frequency of IFs in CBCT imaging is much larger in number and in scope when compared to traditional 2-D imaging. Bondemark et al. [51] and Asaumi et al. [82] reported that incidental findings were respectively identified in only 8.7% and 6.1% of patients when panoramic radiographs were reviewed. Granlund et al. [66] reported an IF frequency of 2.2 IFs per panoramic image, a rate that is consistent with CBCT studies. However, in these three studies, there is no mention of airway, vascular, or cervical vertebral findings, presumably because these anatomic structures are poorly visible in panoramic imaging. Consequently, between 75.0% and 100% of the reported findings were confined within the dento-alveolus [51, 66, 82], a region that dental clinicians should be competent in interpreting. This is in sharp contrast to CBCT studies.

### Clinical significance

Two fundamental matters are apparent upon review of the results, both relating to the clinical implications of the findings. Firstly, of clinical relevance is the percentage of IFs that require further follow-up and/or management from other medical/dental professionals, and secondly is how many IFs alter the orthodontic management of the patient. It must be stated that our study was based solely on radiographic interpretation, as no clinical information or other records were collected and/or considered. Therefore, inference into clinical significance is limited, specifically when relating to airway, sinus, and TMJ findings, as clinical signs/symptoms play an integral role in determining the presence and severity of disease. Thus, the researchers elected to categorize findings as significant, only if they required immediate follow-up. The authors determined by consensus that 11.2% (94/842) of findings required immediate follow-up based on radiographic appearance. The number of findings determined to require immediate follow-up based on anatomic category is listed in Table 4. Only adeno-tonsillar hypertrophy and sinus inflammation reported as severe by the OMFR were included as clinically significant. Examples of the most common significant findings include periapical rarefying osteitis, external root resorption, severe adeno-tonsillar hypertrophy, degenerative TMJ changes, and enlargement of sella turcica. Regarding our sample, it can be argued that the identification of four of the seven findings (Figures 3,4,6,9) recommended for follow-up by our OMFR may have been difficult using only 2-D imaging traditionally utilized in orthodontics.

**Table 4:** **Number of incidental findings reasoned to require follow-up**

Anatomic location	Incidental findings,***n***(%)	Findings requiring follow-up,***n***(%)
Cervical vertebrae	11 (1.3)	1 (0.1)
Dentoalveolar	124 (14.7)	19 (2.3)
Naso-oropharyngeal airway	356 (42.3)	23 (2.7)
Paranasal sinuses	260 (30.9)	22 (2.6)
Surrounding soft/hard tissues	34 (4.0)	11 (1.3)
Temporomandibular joint	54 (6.4)	18 (2.1)
Total	842 (100)	94 (11.2)

It is difficult to discern the impact of these IFs on future orthodontic management in our sample. Several other studies have investigated the impact of CBCT findings on subsequent treatment planning decisions. Based on these, it is suggested that CBCT may provide more reliable information than 2-D images and that the interpretation of CBCT volumes may result in a different diagnosis and/or an alternative treatment plan for specific conditions such as root angulation, root resorption, third molar impaction, and canine impaction [69],[83–87].

Only one study has investigated the impact of CBCT IFs on treatment decisions regarding subsequent orthodontic treatment planning. Drage et al. [20] determined that 45% of IFs required further follow-up, but less than 1% of IFs were likely to influence orthodontic management. However, further investigations are needed to assess the impact of IFs on the management decisions made by clinicians and their impact on subsequent orthodontic treatment.

### Limitations

There are several limitations with this descriptive cross-sectional study. Only a single board-certified OMFR interpreted all CBCT scans. Thus, the interpretation reports are subject to reporter bias, with an unknown possibility of inconsistent diagnoses and errors. The subjective process of placing findings into anatomic categories can lead to differences when comparing studies in the literature. This is common when examining airway versus sinus findings, as some studies combined them into a single group, while they were separated in other studies, leading to either an under- or overestimation of findings for certain anatomic regions. Another inadequacy is that limited clinical and no prior radiographic or histological information was obtained to determine if the identified CBCT findings had been previously detected; this analysis was outside the scope of this study. Also, no clinical correlations of the findings were obtained as this study exclusively evaluated only the image data. Ideally, forthcoming research will investigate the impact of these findings on subsequent orthodontic management in terms of potential alteration of the treatment plant or the need for further multidisciplinary care.

## Conclusions

This study confirms the high occurrence of incidental findings in large field-of-view maxillofacial CBCT scans in an orthodontic population. These findings suggest that the large majority are extragnathic findings, which can be normally considered outside the regions of interest and expertise of many dental clinicians. Specifically, incidental findings in the naso-oropharyngeal and paranasal air sinuses are the most frequent. This underscores the need for comprehensive review of the entire data volume and the requisite to properly document all findings, regardless of the region of interest.

## Authors’ original submitted files for images

Below are the links to the authors’ original submitted files for images. Authors’ original file for figure 1 Authors’ original file for figure 2 Authors’ original file for figure 3 Authors’ original file for figure 4 Authors’ original file for figure 5 Authors’ original file for figure 6 Authors’ original file for figure 7 Authors’ original file for figure 8 Authors’ original file for figure 9
