# Supplementary figures and images for: The frequency and nature of incidental findings in large-field cone beam computed tomography scans of an orthodontic sample
Source: Prog Orthod. 2014 Jun 11;15:37. doi: 10.1186/s40510-014-0037-x (PMC4884029; doi:10.1186/s40510-014-0037-x)

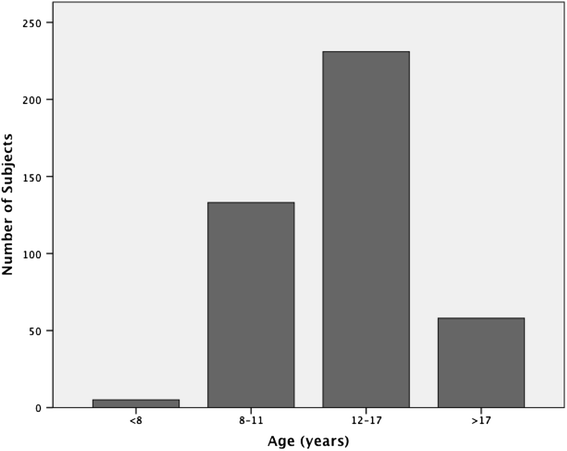

Supplement: Supplementary file 1 — Authors’ original file for figure 1 [file 40510_2014_37_MOESM1_ESM.gif]

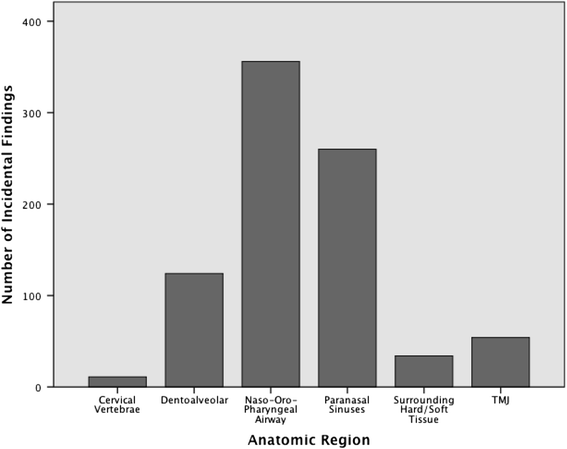

Supplement: Supplementary file 2 — Authors’ original file for figure 2 [file 40510_2014_37_MOESM2_ESM.gif]

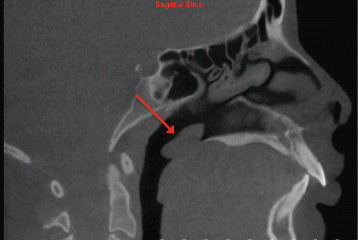

Supplement: Supplementary file 3 — Authors’ original file for figure 3 [file 40510_2014_37_MOESM3_ESM.gif]

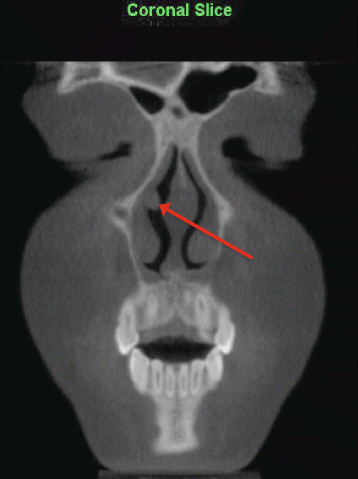

Supplement: Supplementary file 4 — Authors’ original file for figure 4 [file 40510_2014_37_MOESM4_ESM.gif]

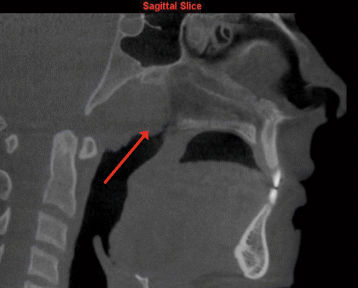

Supplement: Supplementary file 5 — Authors’ original file for figure 5 [file 40510_2014_37_MOESM5_ESM.gif]

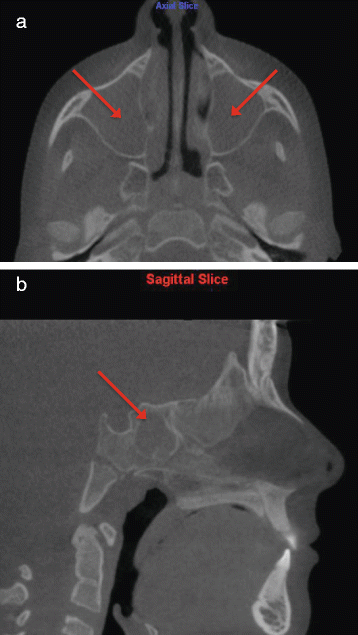

Supplement: Supplementary file 6 — Authors’ original file for figure 6 [file 40510_2014_37_MOESM6_ESM.gif]

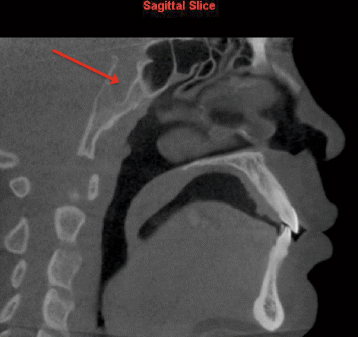

Supplement: Supplementary file 7 — Authors’ original file for figure 7 [file 40510_2014_37_MOESM7_ESM.gif]

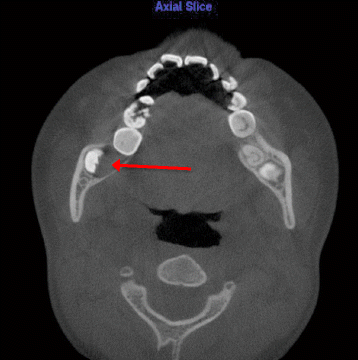

Supplement: Supplementary file 8 — Authors’ original file for figure 8 [file 40510_2014_37_MOESM8_ESM.gif]

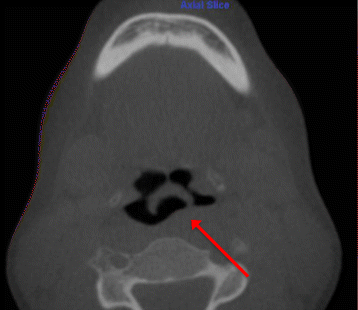

Supplement: Supplementary file 9 — Authors’ original file for figure 9 [file 40510_2014_37_MOESM9_ESM.gif]
